# The safety of exercise for older patients with severe aortic stenosis undergoing conservative management: A narrative review

**DOI:** 10.14814/phy2.15272

**Published:** 2022-04-19

**Authors:** Satoshi Nashimoto, Tatsuro Inoue, Kazuki Hotta, Yuichi Sugito, Susumu Iida, Atsuhiro Tsubaki

**Affiliations:** ^1^ Department of Rehabilitation Niigata Medical Center Niigata Japan; ^2^ 52757 Department of Physical Therapy Niigata University of Health and Welfare Niigata Japan

**Keywords:** activities of daily living, aortic valve stenosis, conservative management, rehabilitation, symptomatic

## Abstract

The incidence of aortic stenosis (AS) increases with age and is a serious problem in an aging society. In recent years, transcatheter aortic valve implantation (TAVI) has been performed widely; however, older patients may be ineligible for TAVI or surgical treatment because of medical ineligibility. Symptom‐based rehabilitation is required for these patients to maintain and improve their physical function and ability to perform activities of daily living. No studies have examined exercise safety for older patients with severe AS who are ineligible for TAVI or surgery. We summarized the safety of exercise for older patients with severe AS, collecting 7 studies on maximal exercise stress tests and 16 studies on preoperative physical examinations. From this review, it may be unlikely that exercise under appropriate management can cause hemodynamic changes, leading to death. However, there were no studies on exercise intervention for older patients with AS who are chosen for conservative management. The optimal exercise intensity for symptomatic older patients with AS undergoing conservative management and the effects of continuous exercise intervention require future study.

## Graphical abstract

We summarized the safety of exercise for older patients with severe AS. From this review, it may be unlikely that exercise under appropriate management can cause hemodynamic changes, leading to death. There were no studies on exercise intervention for older patients with AS who are chosen for conservative management, the optimal exercise intensity and the effects of continuous exercise intervention require future study.

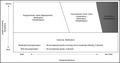

## 1 INTRODUCTION

The incidence of aortic stenosis (AS), which increases with age, is a serious problem in an aging society (Danielsen et al., 2014; De Sciscio et al., 2017; Durko et al., 2018; Eveborn et al., 2013; Nkomo et al., 2009). In asymptomatic patients with severe AS, the risk of sudden death is low (<1%/year). However, once symptoms appear, 3% of patients may die within 6 months and 50% within 2 years (Otto et al., 1997; Pellikka et al., 2005; Rosenhek et al., 2000, 2010). Thus, managing symptomatic patients with severe AS is a critical problem because of improving life expectancy.

Management is determined by symptoms, disease severity, and surgical tolerance (De Sciscio et al., 2017; Durko et al., 2018; Otto et al., 2021; Vahanian et al., 2021). In symptomatic cases, surgical treatment or transcatheter aortic valve implantation (TAVI) is the first choice (Vahanian et al., 2021; Otto et al., 2021). In recent years, TAVI has become popular and is now indicated for some high‐risk patients (Vahanian et al., 2021; De Sciscio et al., 2017; Durko et al., 2018; Iung et al., 2005; Otto et al., 2021). The older patients who also have frailty or comorbidities such as chronic obstructive pulmonary disease, anemia, and other systemic conditions, TAVI are considered in weighing the risk–benefit ratio in an individual patient (Otto et al., 2021). Durko et al. reported that 58.4% of symptomatic patients with AS underwent surgical aortic valve replacement (SAVR), 61.7% of high‐risk patients who had difficulty with SAVR underwent TAVI, and the remaining 38.3% received medical therapy alone (Durko et al., 2018). Patients unable to undergo SAVR or TAVI require rehabilitation to maintain and improve their physical function and ability to perform activities of daily living (ADLs), checking for symptoms such as heart failure (Horstkotte & Loogen, 1998). However, the actual rehabilitation status for older patients with severe AS remains unclear.

**FIGURE 1:**
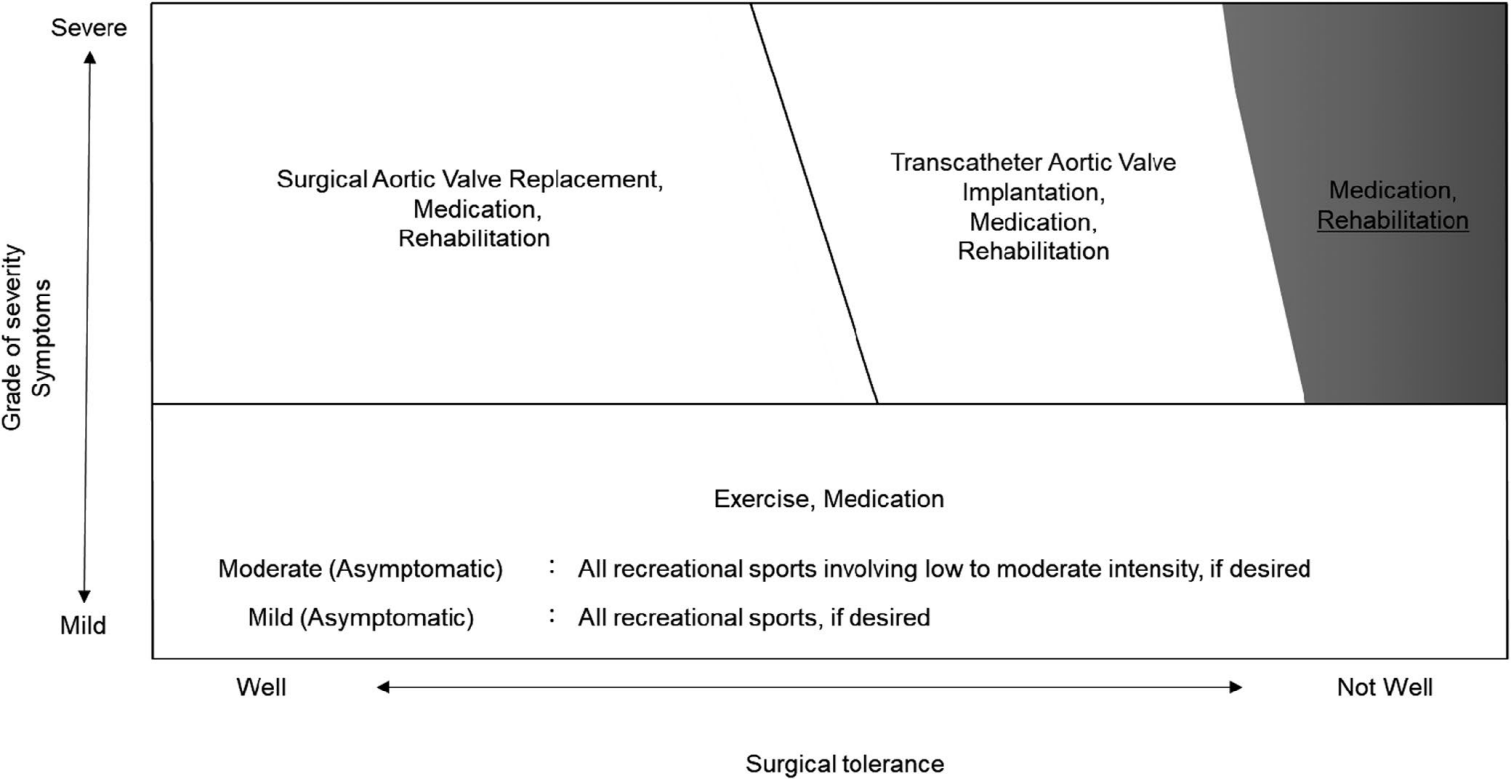
Management of aortic valve stenosis

Improving physical function and ADLs for older patients with severe AS undergoing conservative management should not be overlooked. For asymptomatic patients with mild to moderate AS, participation in recreational sports of low to moderate intensity is recommended for individuals with a left ventricular ejection fraction (LVEF) >50% and good functional capacity (Pelliccia et al., 2021). For symptomatic patients with severe AS, physical frailty is associated with increased mortality, and this association does not vary by treatment type (SAVR or TAVI) or conservative management (Rodríguez‐Pascual et al., 2016). In contrast, patients with symptomatic valvular disease are considered unstable and have activity limitations (Fletcher et al., 2013). Figure 1 summarizes the treatment, exercise, and rehabilitation of patients with AS in their severity and surgical tolerance, but the exercise and rehabilitation of these patients are constrained.

No studies have indicated safe rehabilitation methods for these patients. Evidence on the appropriate exercise intensity and duration to maintain and improve physical function is also lacking. Understanding what is safe for these patients can help cardiologists and physical therapists prescribe suitable exercises. In this review, we summarize the reality and safety of exercise for older patients with severe AS undergoing conservative management.

## 2 MATERIALS AND METHODS

We pooled clinical studies on exercise in subjects, including older patients with severe AS, extracting those in which exercise intervention was performed. We used PubMed (MEDLINE) to search for studies published between 2011 and April 2021, with the following keywords: “aortic valve stenosis,” “conservative,” “exercise,” “physical therapy,” and “rehabilitation.” To review recent studies in older patients with severe AS, we selected the studies published in English 10 years since the PARTNER trial was published in 2010 (Leon et al., 2010). The inclusion criteria for the studies considered in this review were as follows.

- Observational and intervention studies.
- Studies of exercise interventions in the conservative period of aortic valve stenosis.
- Studies involving elderly patients aged 60 years or older.

To clarify the safety of exercise stress in clinically problematic symptomatic cases, we also included studies that conducted preoperative physical examinations. We excluded studies that nonhuman subjects; postoperative exercise interventions; patients aged <60 years; case reports, reviews, protocols, and incidence reports; and those published in languages other than English.

**FIGURE 2:**
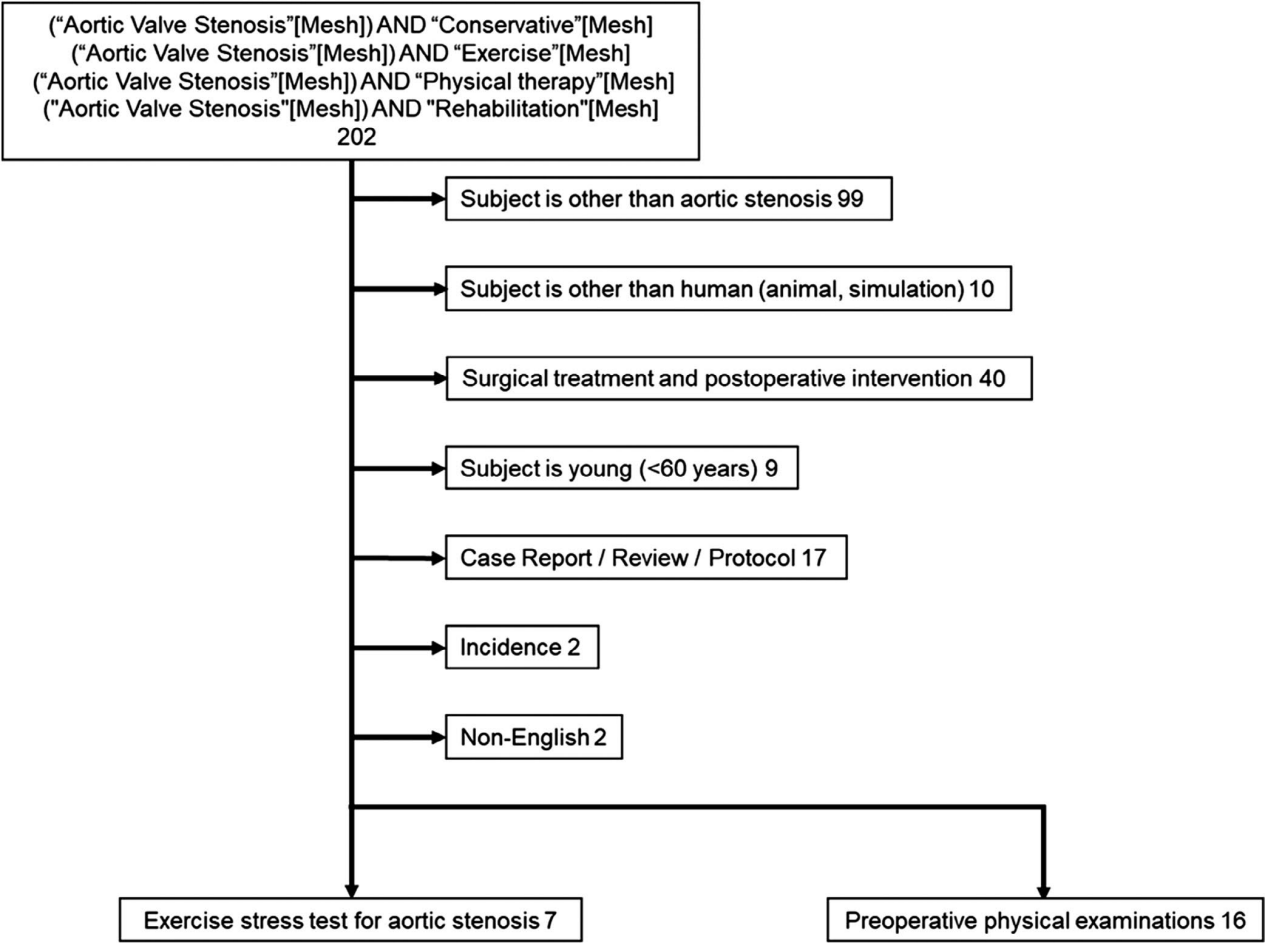
Flowchart of the study selection

One of the authors (S.N.) reviewed the abstracts of these studies for potential inclusion in the study. In this part, preference was given to the inclusion side. If the full text of studies did not meet the inclusion criteria, the study was excluded. Finally, we identified seven studies that performed maximal exercise stress tests; in addition, we identified 16 studies that performed preoperative physical examinations. Figure 2 showed the flowchart of paper selection.

We extracted data on serious adverse events, such as death precipitated by the exercise tests, and symptoms during “exercise” angina, dyspnea, abnormal blood pressure (BP) decrease or elevation, and electrocardiogram changes (nonsustained), arrhythmias (Huded et al., 2018), ST depression by more than 2 mm during testing (Van Le et al., 2016) from the studies retrieved. Abnormal BP response was defined as decrease during testing (Huded et al., 2018), lack of systolic BP increases of at least 20 mmHg (Van Le et al., 2016), a sustained fall in systolic BP at least 20 mmHg below the previous stage or a failure to rise from the baseline level, and peak systolic BP at least 190 mmHg during testing (Saeed et al., 2019). In addition, we investigated patients’ hemodynamic changes.

## 3 RESULTS

In the studies on maximal exercise stress tests, one used the 6‐min walk test (Sigvardsen et al., 2017), two used treadmill tests (Huded et al., 2018; Saeed et al., 2019), two were ergometer interventions in the supine position (one also performed a treadmill test) (Christensen et al., 2016; Lumley et al., 2016), one was a cardiopulmonary exercise test in the upright position (Van Le et al., 2016), and one involved the Valsalva maneuver (Losi et al., 2015). Four included asymptomatic cases (Christensen et al., 2016; Huded et al., 2018; Saeed et al., 2019; Sigvardsen et al., 2017), two included symptomatic cases (Losi et al., 2015; Lumley et al., 2016), and one included both (Van Le et al., 2016). All studies included patients with a preserved LVEF ≥50%.

No studies reported serious adverse events, such as death or symptom worsening precipitated by the exercise stress tests. Dyspnea, arrhythmia, and dizziness during exercise occurred in 18%−67% of the patients. The rate of symptom occurrence was high with the Valsalva maneuver, and no studies reported symptoms during exercise in the supine position (Table 1). Patients with other valvular diseases (Huded et al., 2018; Saeed et al., 2019; Sigvardsen et al., 2017; Van Le et al., 2016), anemia (Saeed et al., 2019), and cases of arthritis that were difficult to perform the exercise test (Saeed et al., 2019) were excluded from each study. Patients with the chronic obstructive pulmonary disease were included in two studies (Sigvardsen et al., 2017; Van Le, 2016).

**TABLE 1:** Exercise stress test for older patients with AS

Authors (year)	Exercise	Sublect (female/male), n	Age (years)	BMI (kg/m²)	Asymptomatic or symptomatic	Echocardiography (rest)				Symptoms during exercise (n, %)	Serious adverse events
						AVA (cm²) or AVAI (cm²/m²)	Peak velocity (m/s)	Mean gradient aortic valve (mmHg)	LVEF (%)		
Huded et al., 2018	Treadmill test	504 (112/392)	66 ± 12	28 ± 5	Asymptomatic	AVA,079 ± 0.2 AVAI, 0.46 ± 0.1 [<0.6]	–	58 ± 18 [>50]	58 ± 4 [>50]	General fatigue: 411 (81.5%) Dyspnea: 42 (8.3%) Abnormal BP response: 25 (5.0%) Angina: 15 (3.0%) Arrhythmia: 8 (5 NSVT) (1.6%) Dizziness: 3 (0.6%)	–
Van Le et al., 2016	CPX upright bicycle ergometer	131 (48/83)	72.1 ± 9.3	26.8 ± 4.0	Asymptomatic and symptomatic	AVA, [<1.3] AVAI, 0.45 ± 0.11	3.92 ± 0.77	38.2 ± 15.3	[>50]	Angina, severe dizziness, discomfort, dyspnea: 25 (19.1%) Abnormal BP response: 35 (26.7%) ST depressions: >2 mm 12 (9.2%)	–
Saeed et al., 2019	Treadmill test	Normotensive, 85 (26/59)	58 ± 14	–	Asymptomatic (Moderate−severe)	AVA, 0.94 ± 0.23	3.7 ± 0.7	35 ± 15	61 ± 8	Abnormal BP response: 23 (27.1%)	–
		Hypertensive, 229 (78/151)	68 ± 10	–		AVA, 0.94 ± 0.2	3.7 ± 0.7	34 ± 13	60 ± 6	Abnormal BP response: 69 (30.1%)	
Sigvardsen et al., 2017	6MWT	Nonperformer, 9 (2/7)	72 ± 9	31.3 ± 6.9	Asymptomatic (Mild−severe)	AVA, 1.14 ± 0.28	3.3 ± 0.6 [>2.5]	25 ± 11	60 (57–68) [>50]	–	–
		Short, <390 m, 37 (14/23)	75 ± 9	27.8 ± 4.0		AVA, 1.01 ± 0.24	3.3 ± 0.6 [>2.5]	28 ± 12	59 (55–64) [>50]		
		Intermediate, 390−465 m, 37 (10/27)	73 ± 7	26.4 ± 4.2		AVA, 0.99 ± 0.35	3.4 ± 0.8 [>2.5]	30 ± 18	57 (55–62) [>50]		
		Long, >465 m, 33 (5/28)	67 ± 8	26.0 ± 3.0		AVA, 1.01 ± 0.32	3.57 ± 0.8 [>2.5]	33 ± 17	60 (55–67) [>50]		
Christensen et al., 2016	Supine cycle ergometer exercise and treadmill test	39 (9/30)	73 ± 7 [>18]	–	Asymptomatic	AVA, 0.83 ± 0.17 [<1.0] AVAI, 0.44 ± 0.09	4.2 ± 0.5 [>3.0]	46 ± 13	66 ± 7 [>50]	–	–
Lumley et al., 2016	Supine bicycle exercise	22 (4/18)	69 ± 8	–	Symptomatic	0.74 ± 0.16 [<1.0]	4.7 ± 0.66 [>4.0]	57 ± 16	59 ± 5 [>50]	–	–
Losi et al., 2015	Performing a Valsalva maneuver	15 (10/5)	65 ± 8	–	Symptomatic	AVAI, 0.46 ± 0.1 [<0.6]	–	43 ± 10	[>50]	Dyspnea: 10 (66.6%)	–

Note

Reported as the number or mean ± SD or median (25%–75% value) [inclusion criteria].

Abbreviations: 6MWT, 6‐min walk test; AS, aortic stenosis; AVA, aortic valve area; AVAI, aortic valve area index; BMI, body mass index; BP, blood pressure; CPX, cardiopulmonary exercise test; LVEF, left ventricular ejection fraction.

The hemodynamics during exercise are shown in Table 2. Aerobic exercises increased the heart rate, systolic BP, and diastolic BP of patients in all studies. Only one study showed a trend toward no change in the LVEF and an increase in the aortic valve peak velocity max, aortic valve mean gradient, pulmonary capillary wedge pressure, and cardiac index during exercise (Christensen et al., 2016). Conversely, when left atrium dilation was apparent, especially when the E/e′ was increased, the pulmonary capillary wedge pressure was high, particularly with exercise (Christensen et al., 2016). Saeed et al. reported that lower peak systolic BP and a rapid early rise in heart rate were associated with symptom occurrence during exercise (Saeed et al., 2019). With the Valsalva maneuver, no change in BP occurred, and there was a trend toward a decreased pressure gradient and cardiac output (Losi et al., 2015). Patients who had dyspnea during the Valsalva procedure had a higher left atrium volume index and delta stroke volume index than those who did not. Many patients in these studies were on medical controls, such as beta blockers, diuretic, calcium channel blockers, angiotensin‐converting enzyme inhibitors, and AII inhibitors (Christensen et al., 2016; Huded et al., 2018; Saeed et al., 2019; Sigvardsen et al., 2017; Van Le et al., 2016).

**TABLE 2:** Hemodynamics during exercise stress test for older patients with AS.

Authors (year)	Exercise	Subject (n)	HR (beats/min)		SBP (mmHg)			SpO₂ (%)		Echocardiography		Other	
			Pretest	Peak		Pretest	Peak	Pretest	Peak	Pretest	Peak	Pretest	Peak
Huded et al., 2018	Treadmill test	504	68 ± 13	136 ± 23		137 ± 18	165 ± 27	–	–	–	–	–	–
Van Le et al., 2016	CPX upright bicycle ergometer	Asymptomatic, 38	–	–		–	–	–	–	–	–	SVi, 35.0 ± 8.5 ml/m²	SVi, 44.9 ± 9.3 ml/m²
		Equivocal symptomatic, 92	–	–		–	–	–	–	–	–	SVi, 31.3 ± 8.7 ml/m²	SVi, 38.9 ± 9.3 ml/m²
Saeed et al., 2019	Treadmill test	Normotensive, 85	76 ± 13	141 ± 25		129 ± 15	158 ± 25	–	–	–	–	–	–
		Hypertensive, 29	77 ± 16	131 ± 24		146 ± 18	169 ± 26	–	–	–	–	–	–
Sigvardsen et al., 2017	6MWT	Short, <390 m, 37	66 ± 11	73 ± 11*		–	–	96 ± 2	97 ± 3*	–	–	–	–
		Intermediate, 390−465 m, 37	64 ± 11	77 ± 13*		–	–	97 ± 2	97 ± 2*	–	–	–	–
		Long >465 m, 33	63 ± 10	75 ± 13*		–	–	96 ± 2	96 ± 3*	–	–	–	–
Christensen et al., 2016	Supine cycle ergometer exercise and treadmill test	LAVi <35 ml/m², 14	64 ± 11	120 ± 23		–	–	–	–	LVEF, 63 ± 8% Peak velocity, 4.0 ± 0.3 m/s AV mean gradient, 40 ± 7 mmHg	LVEF, 62 ± 9% Peak velocity, 4.5 ± 0.3 m/s AV mean gradient, 53 ± 12 mmHg	PCWP, 9 ± 2 mmHg CI, 2.7 ± 0.8 l/min/m²	PCWP, 27 ± 3 mmHg CI, 6.3 ± 1.4 l/min/m²
		LAVi >35 ml/m², 25	65 ± 10	115 ± 16		–	–	–	–	LVEF, 67 ± 6% Peak velocity, 4.4 ± 0.6 m/s AV mean gradient, 49 ± 14 mmHg	LVEF, 67 ± 9% Peak velocity, 4.8 ± 0.7 m/s AV mean gradient, 61 ± 18 mmHg	PCWP, 14 ± 4 mmHg CI, 2.9 ± 0.5 l/min/m²	PCWP, 33 ± 5 mmHg CI, 6.6 ± 1.4 l/min/m²
Lumley et al., 2016	Supine bicycle exercise	22	75 ± 16	117 ± 25		130 ± 19	155 ± 27	–	–	–	–	–	–
Losi et al., 2015	Performing a Valsalva maneuver	15	–	–		133 ± 14	131 ± 14	–	–	AVAi, 0.46 ± 0.1 cm²/m² AV mean gradient, 43 ± 10 mmHg	AVAi, 0.47 ± 0.1 cm²/m² AV mean gradient, 30 ± 9 mmHg	SVi, 44 ± 6 ml/m²	SVi, 32 ± 6 ml/m²

Note

Reported as the mean ± SD.

Abbreviations: AS, aortic stenosis; AVAi, aortic valve area index; CI, cardiac index; CPX, cardiopulmonary exercise test; HR, heart rate; LAVi, left atrium volume index; LVEF, left ventricular ejection fraction; PCWP, pulmonary capillary wedge pressure; SBP, systolic blood pleasure; SVi, stroke volume index.

* Immediately after the test.

Preoperative physical examinations before surgery or TAVI included the 6‐min walk test (Abdul‐Jawad Altisent et al., 2017; DeLarochellière et al., 2015; Eichler et al., 2020; Green et al., 2013; Mok et al., 2013), gait speed (walking 15 feet) (Chauhan et al., 2016; Forcillo et al., 2017; Green, Woglom, Genereux, Daneault, et al., 2012; Green, Woglom, Genereux, Maurer, et al., 2012; Hebeler et al., 2018; Kotajarvi et al., 2017; Rodríguez‐Pascual et al., 2016; Rogers et al., 2018), grip strength (Chauhan et al., 2016; Eichler et al., 2020; Forcillo et al., 2017; Green, Woglom, Genereux, Daneault, et al., 2012; Green, Woglom, Genereux, Maurer, et al., 2012; Hebeler et al., 2018; Kotajarvi et al., 2017; Rodríguez‐Pascual et al., 2016; Rogers et al., 2018), and timed up and go test (Eichler et al., 2020; Schoenenberger et al., 2013, 2018; Stortecky et al., 2012).

No studies reported serious adverse events, such as death or symptom worsening (Table 3). However, in some studies, patients had difficulty walking because of heart failure symptoms. In such cases, the patients were excluded (Abdul‐Jawad Altisent et al., 2017) or analyzed as having a zero walking speed or distance (DeLarochellière et al., 2015; Green, Woglom, Genereux, Daneault, et al., 2012; Green, Woglom, Genereux, Maurer, et al., 2012; Mok et al., 2013). In 16 studies, preoperative physical function affected postoperative life expectancy (Abdul‐Jawad Altisent et al., 2017; Chauhan et al., 2016; Forcillo et al., 2017; Green et al., 2013; Green, Woglom, Genereux, Maurer, et al., 2012; Rodríguez‐Pascual et al., 2016; Rogers et al., 2018; Schoenenberger et al., 2013, 2018; Stortecky et al., 2012), cardiovascular event occurrence (Stortecky et al., 2012), preoperative ADLs dependence (Green, Woglom, Genereux, Daneault, et al., 2012), and postoperative functional decline (Schoenenberger et al., 2013).

**TABLE 3:** Preoperative physical examinations for older patients with AS

Authors (year)	Subject (female/male), n	Age (years)	BMI (kg/m²)	Evaluation period	Echocardiography (rest)			Physical function assessment				Serious adverse events
					LVEF (% or n)	Mean gradient aortic valve (mmHg)	AVA (cm²)	6MWT	Gait speed	Grip strength	TUG	
Abdul‐Jawad et al., 2017	305 (166/139)	79 ± 9	26.5 ± 4.9	Before TAVI	53.2 ± 14.1	40.7 ± 16.8	–	〇				–
DeLarochellière et al., 2015	438 (224/214)	79 ± 8	27 ± 5	Before TAVI	<40%, 72	41 ± 16	0.64 (0.50–0.80)	〇				–
Eichler et al., 2020	249 (147/102)	80.7 ± 5.1	28.0 ± 4.8	Before TAVI	54.5 ± 10.9	44.9 ± 16.5	–	〇		〇	〇	–
Green et al., 2013	Unable to walk, 218 (120/98)	84.6 (79.1–88.9)	26.2 (22.7–30.4)	Before TAVI	55.4 (44.4–61.7)	41.9 (32.5–53.3)	0.63 (0.51–0.76)	〇				–
	Slow walkers, 133 (60/73)	85.9 (81.7–88.5)	26.0 (22.1–30.2)		53.0 (37.6–61.3)	40.4 (33.0–51.2)	0.63 (0.53–0.73)					
	Fast walkers, 133 (44/89)	83.6 (78.3–87.6)	25.5 (22.8–29.0)		57.5 (48.5–64.4)	41.3 (33.3–49.2)	0.66 (0.56–0.79)					
Mok et al., 2013	319 (172/147)	80 ± 8	26.8 ± 5.4	Before TAVI	54 ± 14	40 ± 16	–	〇				–
Rodríguez‐Pascual et al., 2016	Frail, 299 (191/108)	84.0 ± 4.99	–	Before TAVI, SAVR, or medical treatment	>45%, 79.2%	47.8 ± 15.2	–		〇	〇		–
	Non‐frail, 307 (159/148)	81.9 ± 4.3	–		>45%, 87.9%	49.1 ± 15.0	–					
Chauhan et al., 2016	342 (179/163)	81.85 ± 7.49	28.37 ± 6.9	Before TAVI	53.23 ± 14.65	49.94 ± 16.67	–		〇	〇		–
Forcillo et al., 2017	361 (167/194)	82 (76–86)	26.5 (22.7–30.4)	Before TAVI	55 (43–60)	–	–		〇	〇		–
Green, Woglom, Genereux, Maurer, et al., 2012	102 (53/49)	85.9 ± 8.1	24.7 ± 5.6	Before TAVI	50 ± 16 <45%, 28	[>40]	[<0.8]		〇	〇		–
Green, Woglom, Genereux, Daneault, et al., 2012	159 (80/79)	86.2 ± 7.7	24.7 ± 5.6	Before TAVI	48 ± 16	45 ± 15	0.6 ± 0.2		〇	〇		–
Hebeler et al., 2018	468 (220/248)	81.7 ± 7.9	27.5 ± 6.2	Before TAVI	54.2 ± 12.8	–	–		〇	〇		–
Kotajarvi et al., 2017	103 (42/61)	80.6 ± 7.4	30.5 ± 6.5	Before TAVI or SAVR	58.3 ± 12.8	49.0 ± 10.6	0.8 ± 0.2		〇	〇		–
Rogers et al., 2018	544 (277/267)	81 ± 8.4	29 ± 9	Before TAVI	–	–	–		〇	〇		–
Schoenenberger et al., 2013	119 (66/53)	83.4 ± 4.6	26.0 ± 4.7	Before TAVI	50.8 ± 14.2	43.1 ± 15.5	0.6 ± 0.2				〇	–
Schoenenberger et al., 2018	330 (186/144)	83.6 (80.9–86.7)	25.2 (23.0–28.1)	Before TAVI	<35%, 39	41.0 (30.0–52.0)	0.60 (0.50–0.80)				〇	–
Stortecky et al., 2012	100 (60/40)	83.7 ± 4.6	25.6 ± 4.6	Before TAVI	50.5 ± 14.1	43.0 ± 15.9	0.6 ± 0.2				〇	–

Note

Reported as the number or mean ± SD or median (25%–75% value) [inclusion criteria].

Abbreviations: 6MWT, 6‐min walk test; AS, aortic stenosis; AVA, aortic valve area; BMI, body mass index; LVEF, left ventricular ejection fraction; SAVR, surgical aortic valve replacement; TAVI, transcatheter aortic valve implantation; TUG, timed up and go test.

## 4 DISCUSSION

Retrospective studies on the prognosis of severe AS have reported that the most common cardiac‐related deaths are exacerbation of heart failure (25.0%−31.3%) and sudden death (3.9%−13.0%) (Ben‐Dor et al., 2010; Minamino‐Muta et al., 2017; Toyofuku et al., 2017), whereas the relationship between death and exercise stress has not been investigated. From this review, it may be unlikely that exercise under appropriate management can cause hemodynamic changes, leading to death. However, there were no studies on exercise intervention for older patients with AS who are chosen for conservative management. We would like to suggest the possibility of exercise intervention for older conservative patients with AS, by reviewing seven studies in which maximal exercise testing was performed on asymptomatic AS patients and 16 studies in which preoperative evaluations were performed.

### 4.1 Safety of maximal exercise stress tests and physical examinations for older patients with as undergoing conservative management

In this review, most of the studies on maximal exercise stress tests were reported in patients with preserved LVEF and in asymptomatic patients. For asymptomatic patients, exercise testing is recommended to test whether a patient really has symptoms on effort or not (Vahanian et al., 2021; Otto et al., 2021; Saeed et al., 2018). The hemodynamics and symptom occurrence during maximal exercise in patients with reduced LVEF, symptomatic patients, and patients with other complications are unclear. However, there have been no studies of exercise stress and intervention in older patients with symptomatic severe AS undergoing conservative management. Two‐thirds of asymptomatic severe AS patients progress to a symptomatic status within the first 2 years of early follow‐up (1–24 months) (Heuvelman et al., 2012). Thus, we consider the results of this review may suggest, although limited, some precautions for exercise in older patients with symptomatic severe AS undergoing conservative management.

During maximal exercise stress tests, appropriate monitoring should be performed. In studies examining this issue, shortness of breath (Huded et al., 2018; Losi et al., 2015; Van Le et al., 2016), hypotension (Huded et al., 2018; Saeed et al., 2019; Van Le et al., 2016), and chest pain (Huded et al., 2018; Van Le et al., 2016) occurred in up to 60% of patients. Recent international prospective randomized controlled trial, although there were no serious adverse events associated with the maximal exercise stress test, some symptoms have been reported (Banovic et al., 2021). Saeed et al. reported that a rapid early rise in heart rate and a lower peak systolic BP were associated with symptom onset (Chambers et al., 2019; Saeed et al., 2019), suggesting a fall in the cardiac output in early exercise and a subsequent failure to rise. Losi et al. reported a greater left atrial volume at rest and a greater reduction in cardiac output associated with the procedure in patients who experienced shortness of breath compared with those who did not during the Valsalva maneuver, suggesting that reduced diastolic function can reduce the cardiac output during preload reduction actions (Losi et al., 2015). Christensen et al. reported that patients with diastolic dysfunction at rest had higher pulmonary venous pressure (>30 mmHg) during ergometer drive in the supine position (Christensen et al., 2016), which can lead to pulmonary congestion and is associated with shortness of breath (Buller & Poole‐Wilson, 1990). Thus, it is important to monitor BP during exercise and to check resting echocardiographic findings (left atrial volume and diastolic function) when performing exercise interventions.

Hemodynamic changes can occur depending on the exercise modality. Aerobic exercises, such as walking and ergometer, can result in an increased cardiac output (Christensen et al., 2016; Van Le et al., 2016), whereas static exercise using the Valsalva maneuver can result in a decreased output (Losi et al., 2015). These results are similar to those reported in healthy participants (Astrand et al., 1964; Delaney et al., 2020). Hemodynamic changes are different among aerobic exercises, such as walking or muscle strengthening, which can cause shortness of breath. Therefore, appropriate risk monitoring is needed.

Posture may be related to the occurrence of symptoms during exercise. Shortness of breath, hypotension, and chest pain during exercise have been reported in exercises performed in the upright position (e.g., treadmill walking, cardiopulmonary exercise test). Two studies reported no symptoms with exercises performed in the supine position (Christensen et al., 2016; Lumley et al., 2016). Thus, exercises performed in the supine position may reduce the risk of syncope and falls associated with AS and may be a safer method. Further studies on this topic are needed.

During preoperative physical examinations, no studies have reported serious accidents precipitated. Most patients were waiting for TAVI, aged, on average, in their 80s, and some had a reduced LVEF. Several of the 16 studies reported that preoperative physical function affected postoperative outcomes. However, in patients with symptoms such as heart failure, a physical examination such as walking was not performed. When performing physical examinations and exercise intervention for symptomatic patients with AS, it is necessary to manage the underlying disease and medical condition as a prerequisite.

### 4.2 Call for action

Physical therapy is necessary to maintain and improve physical function and to maintain the ability to perform ADLs in older patients with severe AS who are ineligible for surgery or TAVI. However, little evidence exists for exercise or rehabilitation in older patients with severe AS. There is a need to clarify the safety and effects of physical therapy on physical function and ADLs in patients with severe AS in future studies.

The perspective of palliative care cannot be excluded in older patients with severe AS undergoing conservative management (Horstkotte & Loogen, 1998). Goodlin recommended that exercise therapy be continued until the end of life to alleviate fatigue and shortness of breath (Goodlin, 2009). In addition, exercise can reduce anxiety and depression (Silveira et al., 2013), improving quality of life (Zhang et al., 2016). It is necessary to consider how to provide better exercise and end‐of‐life care to these patients while also considering their safety.

### 4.3 Limitation

This review has several limitations. First, we found no reports of continuous exercise interventions in older patients with symptomatic severe AS undergoing conservative management, and exercise intensity and duration to maintain or improve physical function or to acquire the ability to perform ADLs are unknown. Second, the patients in this study were aged 50−80 years, but in clinical settings, health professionals may encounter even older patients. Patients eligible for conservative management may have lower cardiac and physical functions compared with those awaiting surgery or TAVI. Therefore, pilot studies need to examine this population. Safety should be reported based on the definitions of serious adverse event and adverse event (European Medicines Agency, 2016; Ioannidis et al., 2004). Finally, selection and publication bias must be considered.

## 5 CONCLUSIONS

There were no studies on exercise intervention for older patients with AS who are chosen for conservative management. A review of 7 studies in which maximal exercise testing was performed on asymptomatic AS patients and 16 studies in which preoperative evaluations were performed suggests the possibility of providing exercise interventions for symptomatic older patients with severe AS under appropriate management. It is seemingly important to consider exercise for these patients who are in the terminal stages of heart failure to maintain their ADLs and to provide high‐quality end‐of‐life care, and studies of exercise interventions for these patients are needed.

## CONFLICTS OF INTEREST

None.

## AUTHOR CONTRIBUTION

Satoshi Nashimoto: The primary author oversaw writing the main parts of the manuscript. Satoshi Nashimoto, Tatsuro Inoue, Kazuki Hotta, Yuichi Sugito, and Susumu Iida designed the research question. Satoshi Nashimoto, Tatsuro Inoue data collection, analyzed the data. Satoshi Nashimoto, Tatsuro Inoue, Kazuki Hotta, Atsuhiro Tsubaki contributed to critical review of draft manuscripts. All authors have read and approved the final manuscript.
